# 4-1BB Agonism Combined With PD-L1 Blockade Increases the Number of Tissue-Resident CD8+ T Cells and Facilitates Tumor Abrogation

**DOI:** 10.3389/fimmu.2020.00577

**Published:** 2020-04-24

**Authors:** Qiu-xia Qu, Xin-yun Zhu, Wen-wen Du, Hong-bin Wang, Yu Shen, Yi-bei Zhu, Cheng Chen

**Affiliations:** ^1^Department of Respiratory and Critical Medicine, The First Affiliated Hospital of Soochow University, Suzhou, China; ^2^Clinical Immunology Laboratory, The First Affiliated Hospital of Soochow University, Suzhou, China; ^3^Jiangsu Key Laboratory of Clinical Immunology, Soochow University, Suzhou, China

**Keywords:** 4-1BB, PD-L1, tissue-resident CD8+ T cell, immunotherapy, mAb

## Abstract

Although the milestone discovery of immune checkpoint blockade (ICB) has been translated into clinical practice, only a fraction of patients can benefit from it with durable responses and subsequent long-term survival. Here, we tested the anti-tumor effect of combining PD-L1 blockade with 4-1BB costimulation in 3LL and 4T1.2 murine tumor models. Dual treatment induced further tumor regression and enhanced survival in tumor-bearing mice more so than PD-L1 and 4-1BB mAb alone. It was demonstrated that dual anti-PD-L1/anti-4-1BB immunotherapy increased the number of intratumoral CD103+CD8+ T cells and altered their distribution. Phenotypically, CD103+CD8+ T cells expressed a higher level of 4-1BB and PD-1 than their CD103− counterparts. Administration of PD-L1 mAb and 4-1BB mAb further increased the cytolytic capacity of CD103+CD8+ T cells. *In vivo*, CD103−CD8+ T cells could differentiate into CD103+CD8+ progeny cells. In a human setting, more CD8+ T cells differentiated into CD103+CD8+ T cells in the peripheral tumor region of lung cancer tissues than in the central tumor region. Collectively, infiltrated CD103+CD8+ T cells served as a potential effector T cell population. Combining 4-1BB agonism with PD-L1 blockade could increase tumor-infiltrated CD103+CD8+T cells, thereby facilitating tumor regression.

## Introduction

PD-1/PD-L1 blockade immunotherapy has shown potential for many types of cancer, but its clinic efficacy is limited, partly due to the absence of tumoral effector cytotoxic T lymphocytes (CTLs) infiltration (1, 2). Current approaches in cancer immunotherapy seek to restore the immune function of unresponsive T cells by blocking inhibitory signals (3–5). It is also reported that blocking the inhibitory signal while simultaneously providing a costimulatory agonist further increases the presence and immune activities of tumor antigen CTLs (6).

The critical function of CD8+ T cells in cancer control has long been recognized, which was viewed mechanistically in the context of continuous recruitment of effector lymphocyte subsets from circulation (7). Recent evidence showed that a subset of CD8+T cells became resident within tissue environments (8, 9). These tissue-resident T cells were not only distinct from their circulating counterparts, but also have been involved in protective immune responses against foreign pathogens (10, 11). In a tumor setting, emerging evidence reported that there were resident T cells within the tumor microenvironment (12–15). These resident cells could have a prognostic value in human cancers (16–18). However, the exact function of resident T cells in anti-tumor immune response and whether they respond to ICB immunotherapy are still unknown.

Therefore, we hypothesize that the resident T cells could be of great importance in a tumor microenvironment, as well as hold implications for cancer ICB immunotherapy. In this study, we sought to uncover the concurrent targeting of synergy between 4-1BB (agonist) and PD-L1 (blockade) in mice bearing tumors, and revealed that improved immunotherapy efficacy was partially attributed to CD103+CD8+ T cells that were exposed to the combination therapy.

## Materials and Methods

### Mice and Cell Lines

Six- to eight-week-old female C57BL/6 and BALB/c mice were purchased from Vital River Laboratory Animal Technology Co., Ltd (Beijing). All mice were maintained under specific pathogen-free (SPF) conditions. Mice lung carcinoma cell line (3LL) and mice breast cancer cell line (4T1.2) were from the American Type Culture Collection (ATCC, Manassas, VA). 3LL cell was cultured in RPMI1640, and 4T1.2 cell was cultured in DMEM, supplemented with 10% fetal calf serum (FCS), streptomycin (100 mg/mL), L-glutamine (2 mM), penicillin (100 U/mL), and 2-ME (50 mM). The cells were cultured at 37°C with 5% CO_2_.

### Patients

Thirty-seven patients with lung cancer were admitted to the First Affiliated Hospital of Soochow University from 2018 to 2019. All were in the late pathological stage (IIIB–IV), and none of them had received anti-tumor therapy at the time of sample analysis. Clinic information included age, sex, and histological subtype. Of these, 23 were adenocarcinomas, 8 were squamous cell carcinomas, and 6 were small cell carcinoma. This study was approved by ethics committee of the First Affiliated Hospital of Soochow University.

### Sample Collection

Blood (*n* = 10) and malignant pleural effusion (*n* = 7) were obtained from patients diagnosed with lung cancer. For tumor tissue, a bronchoscope was used to attach the lung cancer lesion. To visualize neoplasm under the bronchoscope, a superficial biopsy was performed (*n* = 11). For peribronchial lesions, intratumoral endobronchial ultrasound-guided transbronchial needle aspiration (EBUS-TBNA) with a 22-gauge needle was performed (*n* = 9). This was then aspirated with gentle negative pressure as the needle was inside the tumor lesion. Written informed consent was obtained from all patients.

### Mouse Tumor Experiments

3LL cells were injected subcutaneously into B6 mice, and 4T1.2 cells were injected into the mammary fat pads of BALB/c mice, respectively. The size of tumor was monitored every 2–3 days (19). Tumor bearing mice were randomized into four treatment cohorts: (i) control IgG; (ii) PD-L1 mAb (clone 10F.9G2, BioXCell); (iii) 4-1BB mAb (clone LOB12.3, BioXCell); or (iv) PD-L1 mAb combined with 4-1BB mAb. All antibodies were administered at a dose of 150 μg/mouse through intraperitoneal injection twice per week. Mice were euthanized if the tumor volume reached 2 cm^3^. Survival calculation was according to the day of euthanasia.

4T1.2 metastatic tumor nodules were enumerated on lung after the India ink staining, as reported previously (19). Briefly, India ink solution was injected into lungs through the trachea, and the lungs were stained for 5 min. The lungs were removed and placed in Fekete's solution (10% formalin, 70% alcohol, and 5% acetic acid) for destaining. Tumor nodules in the lung did not absorb ink, which resulted in the tumor nodules remaining white and the normal lung tissue staining black. Then, tumor nodules were counted blindly by two independent investigators (19). During this study, the care of animals was kept in accordance with institution guidelines.

### Analysis of Tumor-Infiltrating Lymphocytes (TILs)

Tumor tissue of humans and mice were dissected and placed in RPMI medium, then disrupted mechanically using scissors, digested with a mixture of DNase I (0.3 mg/ml, Sigma-Aldrich) and Liberase TL (0.2 mg/ml, Roche) in serum-free RPMI 1640 medium for 30 min, and dispersed through a 70-μm cell strainer (Beyotime Biotechnology) (19). Combinations of the following fluorochrome-conjugated antibody (PD-1, 4-1BB, ICOS, Ki-67, CD103) were used for cell staining among defined population of CD45+CD8+ cells. The gating strategy for the analysis was shown in Figure S1. Multi-colored flow cytometry was performed on Cytoflex (Beckman) and data were analyzed with FlowJo software (Tree Star).

Fifty to three hundred milliliters of human malignant pleural effusion was centrifuged at 300 × g for 5 min; afterward, the cells were resuspended in Red Blood Cell Lysis Buffer at room temperature for 5 min. Subsequently, the cells were washed with PBS for FACS.

Fifty microliters of EDTA anti-coagulated whole blood was stained with antibodies at 4°C for 30 min. Then, the cells were incubated with Optilyse C Lysis Solution at 42°C for 10 min. Subsequently, the cells were washed in PBS with a 5-min centrifugation at 300 × g. The supernatant was discarded and the cells were resuspended for FACS.

### Cytotoxicity Assay

As reported previously for similar cytotoxic assays (20, 21), 3LL cells were incubated for 15 min at 37°C with 5 μM CFSE in PBS (CFSE^hi^ 3LL), whereas ID8 reference cells were labeled with 0.5 μM CFSE (CFSE^lo^ ID8), and then washed extensively. CFSE^hi^ 3LL and CFSE^lo^ ID8 were incubated in 24-well plate, respectively. Where indicated, CD103+CD8+ T cells and CD103−CD8+ T cells were added at the ratio of 10:1 to CFSE^hi^ 3LL, respectively, and cultured with rat IgG isotype control or anti-4-1BB mAb (10 μg/ml; BioXCell) or anti-PD-L1 mAb (10 μg/ml; BioXCell) or the combination of anti-4-1BB mAb (10 μg/ml) and anti-PD-L1 mAb (10 μg/ml) in duplicate. After 8 h, CFSE^hi^ 3LL were collected by trypsin and moved into 1.2-mL FACS tubes. Before analysis, CFSE^lo^ ID8 were quantified as the same number of CFSE^hi^ 3LL without T cells, then mixed with each group of CFSE^hi^ 3LL. Killing of 3LL cells by CD103+CD8+ T cells or CD103−CD8+ T cells was calculated as 100% × (1-RF_eff_/RF_ctrl_). RF represents the relative frequency of remaining CFSE^hi^ 3LL cells to CFSE^lo^ ID8 reference cells; RF_ctrl_ represents the RF value in groups without CD103+CD8+ T cells or CD103−CD8+ T cells; and RF_eff_ represents the RF value in groups with CD103+CD8+ T cells or CD103−CD8+ T cells (21). Representative results from one of two performed experiments are shown.

### T Cell Transfer Assay

CD103+CD8+T and CD103−CD8+T cells were sorted from the lymph nodes of untreated 3LL-bearing mice using immunomagnetic beads according to the manufacturer's protocol (MicroBeads, Miltenyi). The lymph nodes were taken from 3LL-bearing mice when the transplanted tumors reached ~5 mm in diameter. The lymph nodes were disrupted mechanically using glass slides in medium, then dispersed through a 70-μm cell strainer. These two groups of CD8+ T cells were then stained with 1 μM CFSE at 37°C *in vitro* and transferred to 3LL-bearing mice through tail vein. After 72 h, lymph node from 3LL-bearing mice was isolated and prepared to single cell suspension respectively. Fluorochrome-conjugated CD8 and CD103 mAb were used for staining among defined population of CFSE+ cells by flow cytometric analysis.

### Immunofluorescent Analysis

Frozen sections (5 μm in thickness) were prepared from tumor tissue, embedded in optimum cutting temperature compound (Sakura Finetek). Then, sections were incubated with 3% BSA for 1 h at 37°C. Following this, they were stained with rat anti-mouse CD8 mAb (1:100) and Armenian hamster anti-mouse CD103 (1:100) overnight at 4°C. After washing with PBS, primary antibodies were detected with Alexa Fluor 594-conjugated goat anti-rat IgG (1:500) and Alexa Fluor 488-conjugated goat anti-Armenian hamster IgG (1:500). After washing, sections were subsequently embedded with DAPI. Sections were scanned using Nikon imaging system (Eclipse Ni-U). Mean fluorescence intensity (OD value) was quantified by ImageJ (22). The cell density at the periphery and center was performed by visually counting cells per field of section.

### Statistical Analysis

Data (mean ± SEM) are representative of independent experiments. We used the two-tailed unpaired Student's *t*-test, Mann–Whitney *U*-test, or the log-rank test (survival studies). A *P* < 0.05 was considered as statistical significant. Asterisk coding is indicated as ^*^P < 0.05; ^**^P < 0.01; n.s. denotes not significant.

## Results

### Anti-4-1BB and Anti-PD-L1 Abs Have a Synergistic Anti-tumor Activity

**Figure 1:**
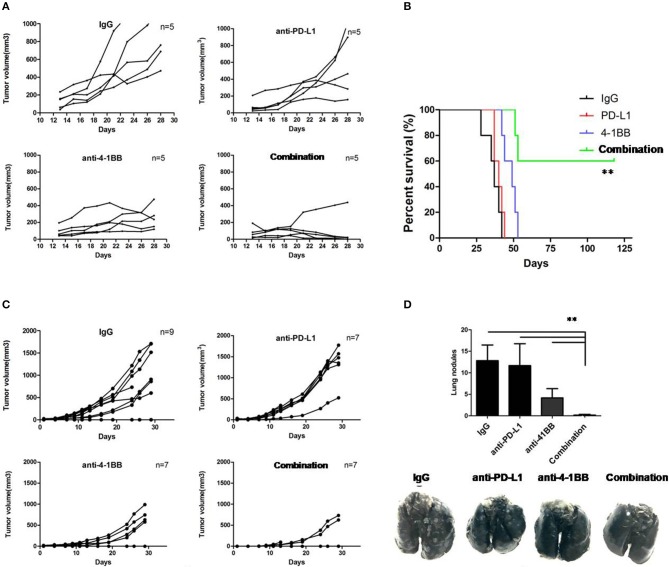
The therapeutic effect of anti-PD-L1 mAb and anti-4-1BB mAb treatment on tumor growth in mice. Individual tumor growth curves showed 3LL tumors grew, but were modest rejected by anti-PD-L1 mAb or anti-4-1BB mAb, with marked tumor delay in combination therapy **(A)**. It also yielded a significantly higher survival rate in dual treatment as compared with the other groups **(B)**. Therapeutic studies were conducted using the 4T1.2 metastatic lung cancer model. In agreement with the 3LL results, combinative therapeutic mice had a significant decrease of primary tumor growth **(C)** and lung metastasis **(D)**. Data were presented as mean ± SEM from independent experiments. **P < 0.01.

To investigate whether it could improve the therapeutic efficacy of ICB in the setting of established tumors, we co-administered anti-PD-L1 mAb and anti-4-1BB mAb to tumor-bearing mice. Four groups of mice were implanted with 3LL cells (2 ×10^5^/mouse). All mice received twice-weekly injection of rat IgG isotype control, anti-PD-L1 mAb, anti-4-1BB mAb, or anti-PD-L1 mAb combined with anti-4-1BB mAb, respectively, beginning at day 10 after tumor inoculation. As shown in Figures 1A,B, anti-PD-L1 mAb alone failed to control 3LL tumor growth, treatment with anti-4-1BB mAb only partially inhibited the growth of tumors, but almost complete inhibition of tumor growth in mice was seen in the combination therapy group (*P* < 0.01). We also observed the survival data until day 125, and mice treated with both anti-4-1BB mAb and anti-PD-L1 mAb experienced the greatest survival benefit as compared with the other groups (*P* < 0.01). Furthermore, we investigated whether the mice (CR) from combined group had generated a memory response to 3LL. We rechallenged them with 3LL cells (4 ×10^5^/mouse), the majority of mice showed the tumor growth again (Figure S2).

Then, therapeutic studies were also conducted in metastatic 4T1.2 tumor model (Figure 1C). Consistent with data above, we found that only mice receivedanti-4-1BB mAb combined with anti-PD-L1 mAb had significantly fewer lung nodules (*P* < 0.01, Figure 1D). The data suggested that combining anti-4-1BB mAb with anti-PD-L1 mAb was sufficient to inhibit the metastatic tumor nodules arising from 4T1.2 cells reaching the lungs.

### Combinatorial Therapy Generates More CD103+ Tumor-Resident T Cells

**Figure 2:**
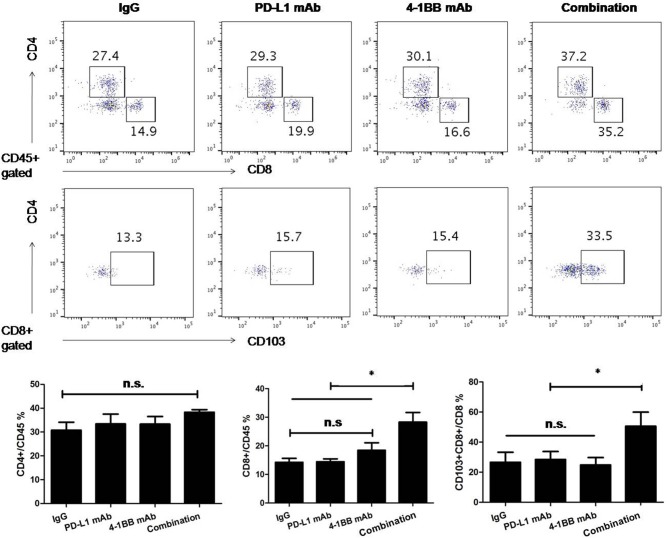
The analysis of TILs was performed by flow cytometry. We empirically defined mAbs treatment schedule, such that tumors were not completely rejected at time of analysis in spite of induction of an effective anti-tumor immune response. Administration of anti-PD-L1 mAb or anti-4-1BB mAb had little or modest effect on the number of infiltrated CD8+ T cells, while the number of infiltrated CD8+ T cells significantly increased in the combinatorial treated group. Notably, there was difference between combinatorial therapy and other groups in terms of the ratio of tumoral CD8+CD103+ T cells. Representative dot plots showed CD4+T, CD8+T, and CD103+CD8+ T cells in tumor microenvirnment. Data were presented as mean ± SEM from independent experiments. *P < 0.05.

To understand the effect of the therapy in the tumor microenvironment and determinate which cell population contributed to tumor growth inhibition and overall survival improvement, we analyzed TILs by flow cytometry. Anti-PD-L1 mAb or anti-4-1BB monotherapy had little or modest impact on the ratio of infiltrated CD8+ T cells, while there was a significant increase in the combinatorial therapy group. Notably, there was a distinction between combinatorial therapy and other groups in terms of the ratio of infiltrated CD103+CD8+ T cells (*P* < 0.05, Figure 2).

**Figure 3:**
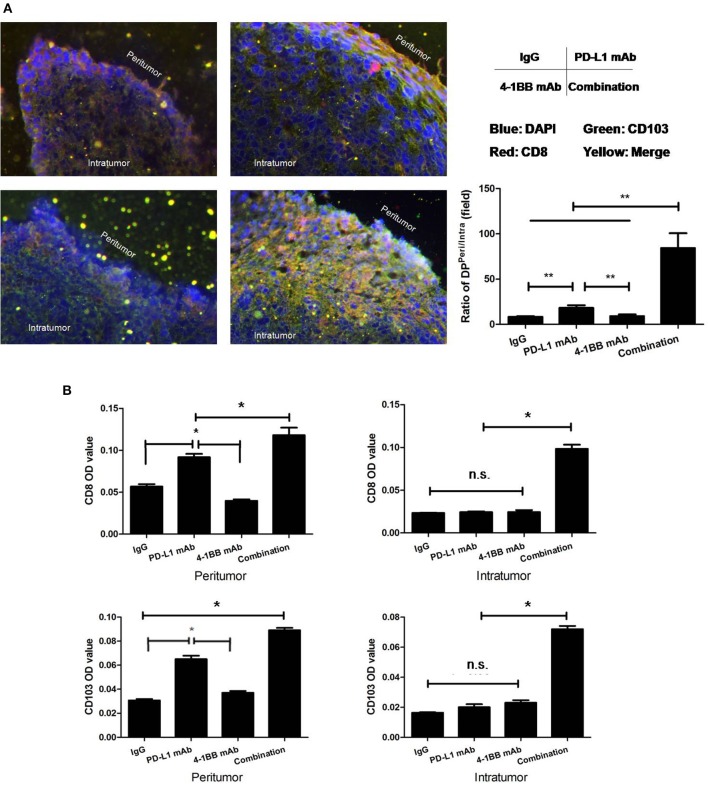
Distribution of tumoral CD103+CD8+ T cells were quantified by immunofluorescent microscope. Serial 3LL transplanted tumor tissue sections were stained with anti-CD8 and anti-CD103 mAb. **(A)** Ratios of CD103+CD8+ T cells “Peritumor” to “Intratumor”; **(B)** the MFI/OD quantification. The results showed that the majority of CD103+CD8+ T cells were located within the peripheral region of the tumor tissue. The dual treatment with anti-PD-L1 mAb and anti-4-1BB mAb promoted CD103+CD8+ T cells to infiltrate toward the central region of tumor tissue. *P < 0.05, **P < 0.01.

To further verify the distribution of tumoral CD103+CD8+ T cells, serial 3LL transplanted tumor sections were stained with anti-CD8 and anti-CD103 mAb, subsequently quantified by immunofluorescent microscope. The data indicated that the density of tumoral CD103+CD8+ T cells varied from mono-therapeutic to combinatorial therapeutic tumor-bearing mice. The majority of CD103+CD8+ T cells were distributed within the peripheral region of tumor tissue, and combining anti-PD-L1 mAb with anti-4-1BB mAb treatment could induce an influx of CD103+CD8+ T cells toward the center of tumor (Figure 3).

### 4-1BB and PD-1 Are Highly Enriched in Tumor-Resident T Cells

**Figure 4:**
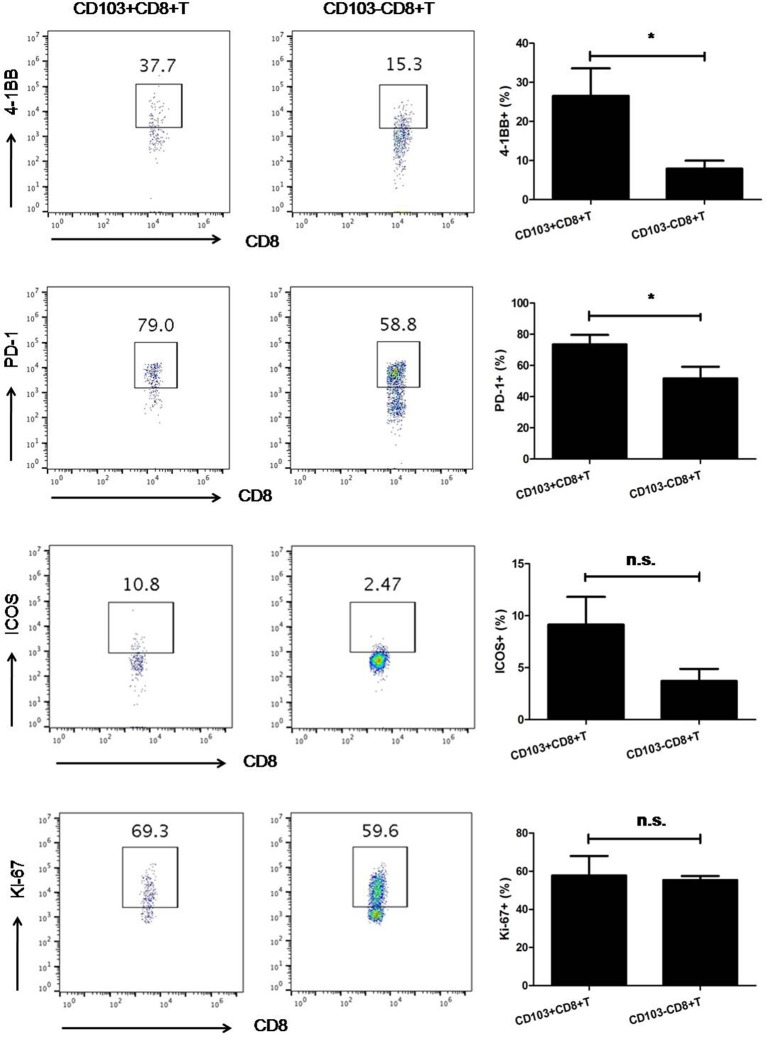
The expression of 4-1BB and PD-1 on CD103−CD8+T cells was higher than that on their CD103−counterparts. Contrary to PD-1 and 4-1BB, CD103+CD8+, and CD103−CD8+ T cells possessed comparable levels of ICOS and Ki-67. Representative flow cytometry plots demonstrated 4-1BB, PD-1, ICOS, and Ki-67 expression on CD103+ and CD103−CD8+T cells subsets. Results were mean ± SEM of independent experiments. *P < 0.05.

Since increased tumoral CD103+CD8+ T cells were observed, the phenotypic characteristics of these T cell subsets were necessary to determine in order to gain insights into their potential role in anti-tumor immune response. Then, we isolated CD103+ tumor-resident T cells from freshly resected 3LL tumor specimens and analyzed the phenotype of these cells by multi-parameter flow cytometry. Interestingly, on average 30% of the CD103+ tumor-resident T cells were 4-1BB positive (*P* < 0.05). Then, we also found CD103+CD8+ T cells expressed higher level of PD-1 than their CD103− counterparts (*P* < 0.05). Contrary to PD-1 and 4-1BB, CD103+ and CD103−CD8+ T cells possessed the comparable level of ICOS and Ki-67 (*P* > 0.05, Figure 4).

### 4-1BB Signaling Synergizes With PD-L1 Blockade to Augment Cytolytic Capacity of Resident T Cells *in vitro*

**Figure 5:**
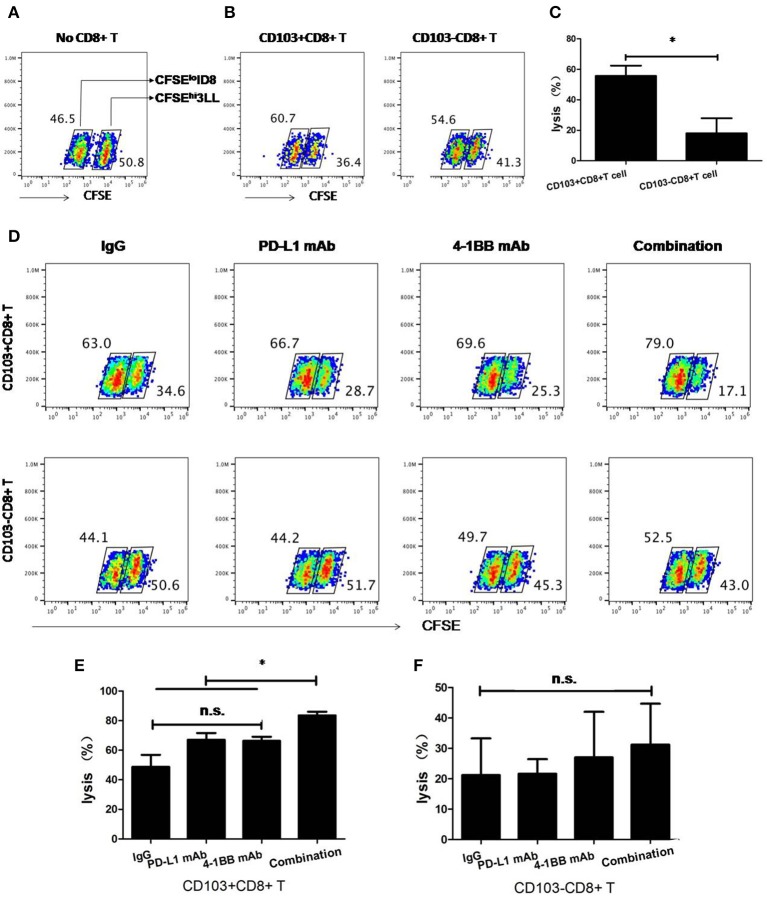
Cytolytic capacity of CD103+ and CD103−CD8+ T cells *in vitro*. 3LL cells were incubated for 15 min at 37°C with 5 μM CFSE (CFSE^hi^ 3LL), whereas ID8 reference cells were labeled with 0.5 μM CFSE (CFSE^lo^ ID8). Where indicated, CD103+ and CD103-CD8+ T cells were added at the ratio of 10:1 to CFSE^hi^ 3LL and cultured with rat IgG isotype, anti-4-1BB mAb, anti-PD-L1 mAb, or the combination of anti-4-1BB mAb and anti-PD-L1 mAb. The numbers within dot plots in **(A,B,D)** indicate RF (%) of CFSE^hi^ 3LL and CFSE^lo^ ID8 cells. Killing of 3LL cells (%) in **(C,E,F)** was calculated as described in section Materials and Methods. Representative results from one of two performed experiments were shown. *P < 0.05.

After that, we compared the cytolytic capacity of CD103+ and CD103−CD8+ T cells *in vitro*. As shown in Figure 5, CD103+CD8+ T cells possessed higher cytolytic capacity than their CD103− counterparts. When anti-PD-L1 blocking mAb or anti-4-1BB agonistic mAb was given alone, there was a weak enhanced cytolytic capacity of CD103+CD8+ T cells. Strikingly, anti-4-1BB mAb combined with anti-PD-L1 mAb could significantly enhance the capacity of CD103+CD8+ T cells to lyse target cells (*P* < 0.05), whereas, cytolytic capacity of CD103−CD8+ T cell was little affected by this treatment. Again, the data clearly highlighted the anti-tumor potential of CD103+CD8+ T cells in the context of combinatorial therapy consisting of 4-1BB agonism and PD-L1 blockade.

### CD103−CD8+ T Cells Convert Into CD103+CD8+ T Cells *in vivo*

**Figure 6:**
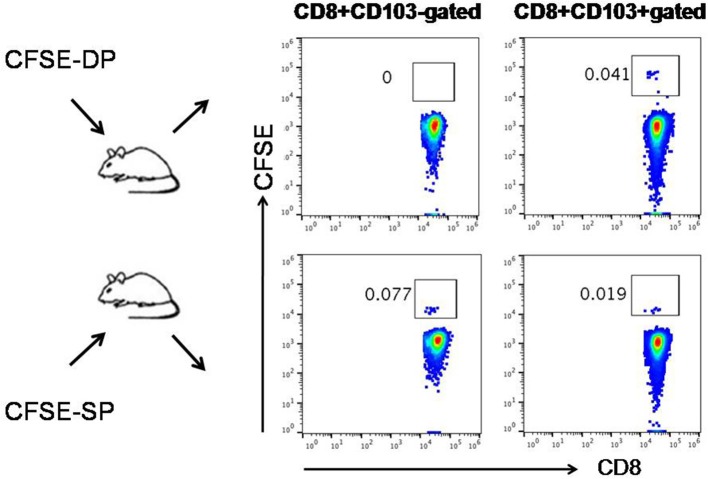
Cross-differentiation between CD103−CD8+ (SP) T cells and CD103+CD8+ (DP) T cells *in vivo*. CD103+ and CD103−CD8+ T cells isolated from untreated tumor-bearing mice were labeled with CFSE and then transferred into mice, respectively. On day 3 post-transfer, the transferred CFSE+SP T cells (CFSE-SP) differentiated into DP T cells, whereas the transferred CFSE+DP T cells (CFSE-DP) failed to differentiate into SP T cells. The numbers of gated cells showed whether CD103^+/−^ could cross-differentiate instead of the percentage numbers of CFSE+ cells converted.

Next, we sought to determine whether CD103+CD8+ T cells could reciprocally convert into CD103−CD8+ T cells *in vivo*. We sorted CD103+ and CD103−CD8+ T cells from untreated tumor-bearing mice and labeled them with CFSE, then transferred these cells into mice (Figure S3). On day 3 post-transfer, it was found that the transferred CFSE+CD103−CD8+ T cells could differentiate into CD103+ T cells, whereas the transferred CD103+CD8+ T cells failed to differentiate into CD103−T cells (Figure 6). These results suggested that CD103−CD8+T cells may serve as progenitors that further differentiate into CD103+ progeny cells. The newly differentiated cells might possess stronger immune function than the cells that were negative for CD103 expression in tumor microenvironment.

### Determination of CD103+ Tumor-Resident T Cells in Human Lung Cancer

CTLs must infiltrate the tumor tissue and interact with the cancer cells to initiate immune response. As an interventional pulmonology, neoplasm-superficial biopsy and intratumoral EBUS-TBNA allowed for a valuable assessment of peripheral and central immunological features in the tumor microenvironment, respectively. Since a concern with EBUS-TBNA is its potential contamination with blood, we also evaluated the circulating T cells to predict how well EUBS-TBNA samples represented the composition of tumor tissue.

**Figure 7:**
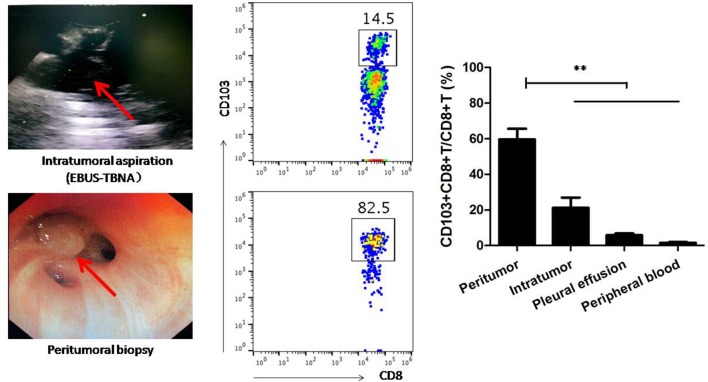
CD103+CD8+ T cells from neoplasm-superficial biopsy, intratumoral EBUS-TBNA, pleural effusion, and peripheral blood of patients with lung cancer were analyzed using flow cytometry. Neoplasm-superficial biopsied tissue and intratumoral EBUS-TBNA tissue represented the periphery and center of lung cancer tissue, respectively. The ratio of CD103+CD8+T cells to CD8+ T cells from neoplasm-superficial sample was obviously more than that from EBUS-TBNA sample, peripheral blood and malignant pleural effusion. Representative flow cytometric plots showed CD103+CD8+T cells in samples. Results are mean ± SEM of independent experiments. **P < 0.01.

To address this, CD103+CD8+ T cells from neoplasm-superficial biopsy and intratumoral EBUS-TBNA in patients with lung cancer were analyzed using flow cytometry. We also investigated the frequency of CD103+CD8+ T cell in peripheral blood and malignant pleural effusion. As shown in Figure 7, the ratio of CD103+CD8+T cells to CD8+ T cells from neoplasm-superficial samples (59.64 ± 6.02%) was obviously more than that from EBUS-TBNA samples (21.24 ± 5.68%, *P* < 0.01), peripheral blood (5.83 ± 0.95%, *P* < 0.01), and malignant pleural effusion (1.50 ± 0.45%, *P* < 0.01), which indicated more CD8+ T cells differentiated into CD103+CD8+ T cells in peripheral tumor microenvironment and less within the center of tumor.

## Discussion

The dysfunction of T cells mediated by the tumor microenvironment is a defining feature of many cancers (23). In a melanoma clinic trial, a high number of infiltrated T cells was a biomarker for clinic responses to anti-PD-1 antibody therapy (24). An alternative to the ICB approach for the reactivation of anti-tumor immune responses is to deliver costimulatory signals of T cells (6).

As a lymphocyte costimulatory receptor, 4-1BB possessed the ability to promote survival, expansion, and enhance effector function of activated T cells, as well as further generate immunologic memory (25, 26). It is plausible that using 4-1BB mAb could potentially augment anti-tumor immunity. For instance, synergistic effects on tumor control for 4-1BB agonist with the PD-1 or CTLA-4 blockade in the human and animal setting were found (27, 28). Combination of 4-1BB agonist and PD-1 antagonist could promote anti-tumor effector/memory response of CD8 T cells in a poorly immunogenic B16 model (29) and ovarian cancer model (30). Anti-4-1BB mAb treatment also improved the therapeutic response to CTLA-4 mAb in colon cancer models (31).

In this work, we demonstrated that combining anti-PD-L1 mAb with anti-4-1BB mAb treatment resulted in a significant tumor abrogation in 3LL model and a decrease of lung MT in 4T1.2 model, whereas 4-1BB agonism or PD-L1 blockade alone had none or only modest effects. Thus, analysis of fresh TIL would not only contribute to investigating the tumor-specific CTL function, but also provide insights into the special CD8+ T cell subsets in a tumor microenvironment.

Resident T cells are a recently identified subset of T cells specialized within tissues. In a tumor microenvironment, CD103 could interact with E-cadherin on the cancer cells, and further promote CCR5 recruitment at the immune synapse formed between T cells and cancer cells (32). A higher accumulation of resident T cells in tumors correlates with better prognosis in cancer patients, and the T cells population are necessary for producing optimal immune control of solid tumors (12–18). However, the fundamental characteristics underlying resident T cells in the tumor microenvironment still remain, and the functional involvement of resident T cells in the context of ICB are poorly investigated.

Here, it was observed that dual anti-PD-L1/anti-4-1BB immunotherapy not only generated a potent CD8+ T cell response, but also promoted intratumoral resident T cell (CD103+CD8+ T cells) infiltration. These results implied that 4-1BB agonism could cooperate with PD-L1 blockade to regulate immune response against resident T cells in the tumor. Furthermore, by immunofluorescent analysis, it was indicated that resident T cells educated by dual treatment effectively penetrated toward the tumor center. We also analyzed the distribution of resident T cells in human lung cancer tissue. Neoplasm-superficial biopsy and intratumoral EBUS-TBNA proved a valuable method to assess the immunological features of the peripheral and central tumor microenvironment respectively. Interestingly, resident T cells were abundantly distributed in periphery of human lung cancer tissue. Taken together, human data were consistent with mice, which showed the probable influx of resident T cells from the periphery to the tumor center. As reported previously, the intra-epithelial location of resident T cells was also found in colorectal (13), bladder (15), and gut (33) cancers. As CD103 contributed to T cell recruitment within epithelial regions, it was believed that CD103 delineated a higher activated T cell subset that could kill more cancer cells than their CD103−counterparts. Generally, penetration of resident T cells toward intratumoral compartment was engaged to anti-tumor immune response.

In addition to the analysis of number of infiltrated CD103+CD8+ T cells in TME, it was also shown that CD103+CD8+ T cells were not only associated with high expression of 4-1BB, but more importantly, with T cell exhaustion marker PD-1. We also noticed that a few of CD103+CD8+ and CD103−CD8+ T cells co-expressed PD-1 and 4-1BB (Figure S4). Based on the profile of 4-1BB and PD-1 on the CD103+CD8+ T cells, it was believed that PD-1 blockade combined with 4-1BB agonism provided strong rescue of exhausted CD8+T cell. It was suggested that the CD103+CD8+ T cells may be the candidate for adjuvant therapy with antibodies targeting 4-1BB or PD-L1/PD-1. As tumor-reactive T cells could be defined by CD103, it may represent a potential biomarker in the context of ICB therapy.

As supported, anti-4-1BB mAb combined with anti-PD-L1 mAb significantly increased the capacity of CD8+CD103+ T cells to kill tumor cells rather than CD8+CD103− T cells, which signified that PD-1 and 4-1BB can deliver selective costimulatory signals to CD103+CD8+ T cells. Based on findings above, we hypothesized that 4-1BB engagement would target resident T cells, while anti-PD-L1 mAb would restore the function of anergized resident T cells. Additionally, such an approach was limited by the little data about fresh TILs from human setting and knowledge of tumor antigen specificity of resident T cells. In addition to PD-1 and 4-1BB, ICOS and Ki-67 was further compared between CD103+ and CD103−CD8+ T cells. Contrary to PD-1 and 4-1BB, CD103+ and CD103−CD8+ T cells possessed the comparable level of ICOS and Ki-67. Therefore, it was required to make deeper characterizations of the CD103^+/−^ CD8+ T cells beyond immune molecules.

In human setting, PD-1 and 4-1BB was compared between CD103+ and CD103−CD8+ T cells. Although CD103+CD8+ T cells expressed a higher level of PD-1 than CD103−CD8+ T cells, they both expressed low level of 4-1BB (Figure S5). Loss the 4-1BB expression on CD8+ T cells would influence the efficacy of immunotherapy.

In NSCLC, CD103+CD8+ T cells has been regarded as tissue-resident memory T cells (14). In other cancers, CD103+CD8+ T cells were of heterogeneous memory phenotypes (34, 35). In present study, although the immunomodulatory effects of therapy suggested a potential for PD-L1 blockade synergized with 4-1BB agonisim, this has yet to be proven in induction of anti-tumor memory immune response.

With regards to phenotype of resident T cells, other studies have suggested that up-regulation of CD103 is mainly the result of TCR signaling upon contact with cancer cells (36). It has also been well-described that CD103 expression could be induced by TGFβR1 activation (37). Consequently, a TGF-β-enriched tumor microenvironment is required for the optimal expression of CD103 (38). Here, using amCFSE-label assay, we determinated the phenotypical convertion between CD103+ and CD103– T cells *in vivo*. It was suggested that transferred CD103−CD8+ T cells differentiate into CD103+CD8+ T cells, whereas the transferred CD103+CD8+ T cells fail to differentiate into CD103−CD8+ T cells. These data indicated that CD103−CD8+ T cells may exert as the progenitors that further differentiate into CD103+ progeny. We further analyzed the level of TGF-β in TME of 3LL model by IHC. In line with others, TGF-β was rich in tumor microenvironment, and monotherapy or combinational therapy could not inhibit the level of TGF-β in tumor tissue (Figure S6). It was suggested that the presence of CD103+CD8+ T cells is derived not only from intrinsic stimulatory conditions favoring the induction of CD103+ on T cells, but also partially due to the concurrent PD-1 blockade and 4-1BB agonism.

Taken together, our findings provided a preclinical strategy to apply 4-1BB agonist combined with PD-L1 blockade to improve the efficacy of cancer immunotherapy. Furthermore, resident T cells was found to exert an important role in the anti-tumor immune response. Hopefully, manipulation of the T cells subset would be explored to improve current cancer immunotherapy approaches (39–41).

## Data Availability Statement

All datasets generated for this study are included in the article/Supplementary Material.

## Ethics Statement

The animal study was reviewed and approved by the ethics committee of The First Affiliated Hospital of Soochow University. The studies involving human participants were reviewed and approved by the ethics committee of The First Affiliated Hospital of Soochow University. The patients/participants provided their written informed consent to participate in this study.

## Author Contributions

QQ and CC designed and performed experiments, analyzed data, and prepared the manuscript. XZ, WD, HW, and YS performed experiments and analyzed data. YZ provided important reagents.

## Conflict of Interest

The authors declare that the research was conducted in the absence of any commercial or financial relationships that could be construed as a potential conflict of interest.

## Supplementary Material

Figure S1 The gating strategy for flow cytometry analysis. CD4+ T cells and CD8+ T cells were gated from CD45+ T cells. Representative flow cytometric plots were from spleens and tumor tissue in tumor-bearing mice.

Figure S2 The CR mice in the combined group (anti-4-1BB mAb and anti-PD-L1 mAb) developed tumors thereafter upon rechallenge. The CR mice were inoculated subcutaneously with 3LL cells on the 114th day. Individual tumor growth curves showed 3LL tumors growth.

Figure S3 The identification of purified CD103+CD8+ T cells **(A)** and CD103−CD8+ T cells **(B)**. CD8+ T cells were taken from draining lymph nodes of untreated 3LL-bearing mice when the implanted tumor reached ~5 mm in diameter.

Figure S4 The co-expression of PD-1 and 4-1BB on CD103−CD8+ T cells and their CD103−counterparts. Representative flow cytometry plots demonstrated 4-1BB and PD-1 expression on CD103+CD8+ and CD103−CD8+T cells subsets from mouse tumor models.

Figure S5 The expression of PD-1 and 4-1BB on CD103+CD8+ and CD103−CD8+ T cells subsets from tumor tissue of lung cancer patients. CD103+CD8+ T cells expressed higher level of PD-1 than their CD103−counterparts. There was low expression of 4-1BB on both of the two cell populations. Results are mean ± SEM of independent experiments.

Figure S6 Immunochistochemical staining microphotographs (×400) of TGF-β in 3LL transplanted tumors. PBS instead of the primary antibody was performed in negative controls. The immunopositivity for TGF-β were defined semiquantitatively in terms of the following criteria. Category A (intensity of immunostaining) was scored using the following criteria: 0, negative; 1, weak; 2, moderate; 3, strong. Category B (percentage of immunoreactive cells) was scored using the following criteria: 0 (0–5%); 1 (5–25%); 2 (26–50%); 3 (51–75%); and 4 (76–100%). The calculation of final scores was multiplying the scores of categories A and B in the same section. Final scores ranged from 0 to 12: 0–2 (–); 3–4 (+); 5–8 (++); 9–12 (+++).
