# Supplementary figures and images for: 4-1BB Agonism Combined With PD-L1 Blockade Increases the Number of Tissue-Resident CD8+ T Cells and Facilitates Tumor Abrogation
Source: Front Immunol. 2020 Apr 24;11:577. doi: 10.3389/fimmu.2020.00577 (PMC7193033; doi:10.3389/fimmu.2020.00577)

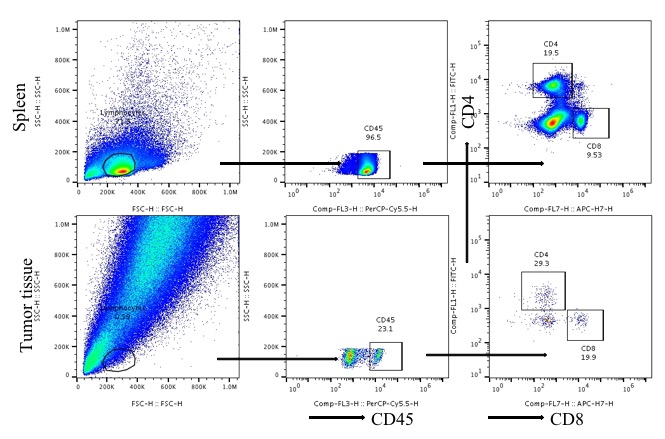

Supplement: Figure S1 — The gating strategy for flow cytometry analysis. CD4+ T cells and CD8+ T cells were gated from CD45+ T cells. Representative flow cytometric plots were from spleens and tumor tissue in tumor-bearing mice. [file Image_1.JPEG]

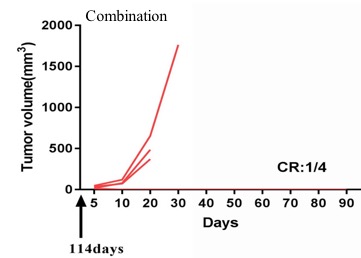

Supplement: Figure S2 — The CR mice in the combined group (anti-4-1BB mAb and anti-PD-L1 mAb) developed tumors thereafter upon rechallenge. The CR mice were inoculated subcutaneously with 3LL cells on the 114th day. Individual tumor growth curves showed 3LL tumors growth. [file Image_2.JPEG]

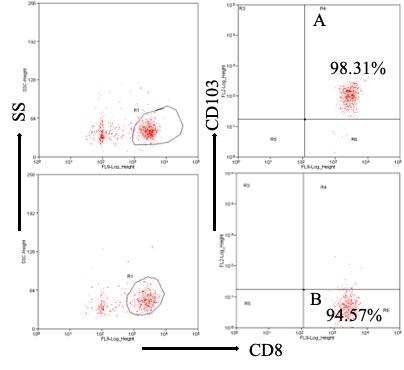

Supplement: Figure S3 — The identification of purified CD103+CD8+ T cells (A) and CD103−CD8+ T cells (B). CD8+ T cells were taken from draining lymph nodes of untreated 3LL-bearing mice when the implanted tumor reached ~5 mm in diameter. [file Image_3.JPEG]

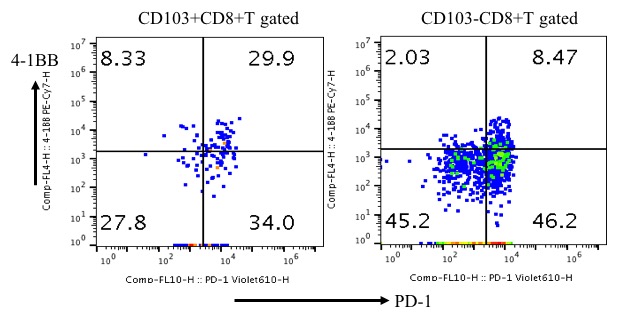

Supplement: Figure S4 — The co-expression of PD-1 and 4-1BB on CD103−CD8+ T cells and their CD103−counterparts. Representative flow cytometry plots demonstrated 4-1BB and PD-1 expression on CD103+CD8+ and CD103−CD8+T cells subsets from mouse tumor models. [file Image_4.JPEG]

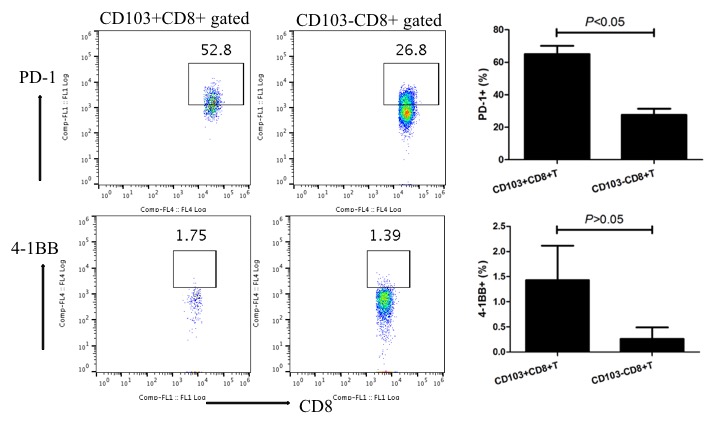

Supplement: Figure S5 — The expression of PD-1 and 4-1BB on CD103+CD8+ and CD103−CD8+ T cells subsets from tumor tissue of lung cancer patients. CD103+CD8+ T cells expressed higher level of PD-1 than their CD103−counterparts. There was low expression of 4-1BB on both of the two cell populations. Results are mean ± SEM of independent experiments. [file Image_5.JPEG]

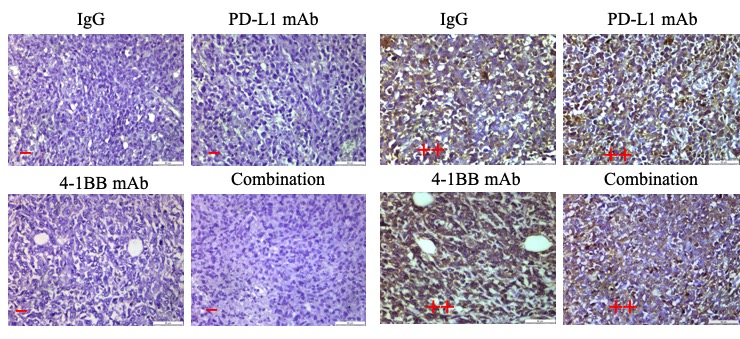

Supplement: Figure S6 — Immunochistochemical staining microphotographs (×400) of TGF-β in 3LL transplanted tumors. PBS instead of the primary antibody was performed in negative controls. The immunopositivity for TGF-β were defined semiquantitatively in terms of the following criteria. Category A (intensity of immunostaining) was scored using the following criteria: 0, negative; 1, weak; 2, moderate; 3, strong. Category B (percentage of immunoreactive cells) was scored using the following criteria: 0 (0–5%); 1 (5–25%); 2 (26–50%); 3 (51–75%); and 4 (76–100%). The calculation of final scores was multiplying the scores of categories A and B in the same section. Final scores ranged from 0 to 12: 0–2 (–); 3–4 (+); 5–8 (++); 9–12 (+++). [file Image_6.JPEG]
